# Latent Symptom Profiles in Adolescents With Major Depressive Disorder: Subjective Sleep Disturbance and Biopsychosocial Correlates

**DOI:** 10.1155/da/9254081

**Published:** 2026-07-24

**Authors:** Jianyu Liu, Xinyi Cao, Mengmeng Yang, Yin Lin, Yan He

**Affiliations:** ^1^ School of Public Health, Zhengzhou University, Zhengzhou, Henan, China, zzu.edu.cn; ^2^ School of Public Health, Hainan Medical University, Haikou, Hainan, China, hainmc.edu.cn; ^3^ Child and Adolescent Psychiatry, Hainan Anning Hospital (Hainan Mental Health Center), Haikou, Hainan, China

**Keywords:** adolescents, latent profile analysis, major depressive disorder, subjective sleep disturbance, trauma/stressor exposure

## Abstract

**Background:**

Adolescent major depressive disorder (MDD) is clinically heterogeneous, with sleep disturbance often emerging as a prominent but variably expressed symptom dimension. This study aimed to identify latent symptom profiles in drug‐naïve adolescents with MDD and examine their associations with recorded trauma/stressor exposure and neuroendocrine markers.

**Methods:**

This cross‐sectional study included 711 drug‐naïve adolescents with MDD. Latent profile analysis (LPA) was conducted using 16 symptom indicators derived from the Hamilton depression rating scale (HDRS), Hamilton anxiety rating scale (HAMA), and Pittsburgh sleep quality index (PSQI). Multinomial logistic regression examined associations of recorded trauma/stressor exposure and neuroendocrine markers with profile membership.

**Results:**

A three‐profile solution was retained: low overall symptoms (Profile 1; *n* = 200, 28.1%), PSQI‐elevated subjective sleep disturbance (Profile 2; *n* = 215, 30.2%), and high overall symptoms (Profile 3; *n* = 296, 41.6%). Recorded family trauma/stressor exposure and violent incident exposure were associated with higher odds of Profile 3 membership relative to Profile 1, and these associations remained significant after neuroendocrine markers were added. Higher standardized testosterone (T) showed a modest association with Profile 3 membership, whereas FT3 showed only a marginal association. However, adding neuroendocrine markers did not significantly improve model fit beyond trauma‐related variables.

**Conclusion:**

LPA identified three clinically interpretable symptom profiles in adolescent MDD, including a profile characterized primarily by elevated PSQI‐assessed subjective sleep disturbance. Recorded trauma/stressor exposure was associated with the high overall symptom profile, whereas neuroendocrine markers showed limited incremental value. These findings highlight multidimensional symptom profiling and trauma assessment in adolescent depression, while suggesting that neuroendocrine findings should be considered exploratory.

## 1. Introduction

Major depressive disorder (MDD) is a major contributor to adolescent disability and is associated with impaired social and academic functioning, nonsuicidal self‐injury, and suicide risk [1, 2]. Epidemiological studies indicate that 15%–20% of adolescents experience a depressive episode by age 18, with a substantial proportion developing severe or recurrent illness requiring intensive clinical care [3, 4].

Adolescent MDD is clinically heterogeneous rather than a single, homogeneous condition [5]. Traditional variable‐centered approaches may be insufficient for identifying distinct symptom constellations within heterogeneous patient populations. Clinical presentations can range from predominant affective symptoms and anxiety‐related features to prominent somatic complaints, including sleep disturbances [6]. In particular, sleep problems frequently co‐occur with depressive symptoms in adolescents but may also represent a relatively distinct and clinically meaningful dimension of illness expression. Prior evidence suggests that somatic and sleep‐related symptoms may influence the illness course, functional impairment, and clinical complexity in adolescent depression [7, 8]. Therefore, characterizing multidimensional symptom profiles may clarify clinical heterogeneity in adolescent MDD and inform more individualized assessment and intervention planning.

Latent profile analysis (LPA) provides a person‐centered statistical approach for identifying unobserved profiles based on patterns of symptom presentation [9, 10]. Compared with conventional severity‐based classifications, LPA can capture clinically meaningful configurations of depressive, anxiety, and sleep‐related symptoms that may not be apparent in variable‐centered analyses. However, studies applying LPA to systematically characterize symptom heterogeneity among drug‐naïve hospitalized adolescents with MDD remain limited. This gap is clinically important because medication exposure and illness chronicity may confound symptom expression, particularly in relation to sleep and neurovegetative symptoms. Moreover, the external correlates of latent symptom profiles, including trauma/stressor exposure and neuroendocrine markers, are not yet well understood in this population.

Trauma/stressor exposure is a major biopsychosocial factor associated with the onset, severity, and course of psychiatric disorders in adolescence [11, 12]. Different forms of trauma/stressor exposure may contribute to distinct clinical presentations through alterations in stress‐response systems, emotion regulation, threat processing, and neurodevelopmental pathways. In adolescents with MDD, trauma may be particularly relevant for distinguishing subgroups with higher overall symptom severity. However, prior research has often examined trauma/stressor exposure in relation to overall depressive severity rather than data‐driven symptom profiles. Whether specific trauma‐related experiences are differentially associated with latent profiles of adolescent MDD remains insufficiently characterized.

Neuroendocrine systems may also contribute to symptom heterogeneity in adolescent depression. Dysregulation involving sex hormones [13] and thyroid hormones [14] has been widely reported in depressive disorders. Estradiol (E2) and testosterone (T) are implicated in neural circuits underlying emotion regulation, cognition, and social behavior [15, 16]. Adolescence is characterized by substantial pubertal hormonal changes that may influence affective and neurocognitive development [17–19]. Early‐life adversity has been linked to alterations in neuroendocrine networks, suggesting that hormonal variation may represent one pathway through which adverse experiences are biologically embedded [20, 21]. Nevertheless, the extent to which single‐time‐point neuroendocrine markers can differentiate clinically meaningful symptom profiles in adolescent MDD remains uncertain.

Existing studies have largely focused on isolated associations among trauma/stressor exposure, neuroendocrine markers, and overall depression severity [22]. Moving beyond global severity, distinct neuroendocrine axes may be differentially related to specific psychobiological domains. For example, the hypothalamic–pituitary–thyroid (HPT) axis plays a central role in cellular metabolism, and thyroid hormone alterations have been associated with somatic and cognitive symptoms such as psychomotor retardation, anergia, and fatigue [23, 24]. Similarly, gonadal hormones may be relevant to stress modulation and affective regulation, with prior studies linking altered sex hormone levels to stress reactivity and affective dysregulation [25, 26]. However, whether such neuroendocrine variations provide incremental explanatory value beyond trauma‐related factors in differentiating latent symptom profiles remains unclear.

To address these gaps, the present study investigated latent symptom profiles in 711 drug‐naïve hospitalized adolescents with MDD using symptom indicators derived from clinician‐rated depressive and anxiety scales and self‐reported sleep assessments. The study had three objectives. First, we aimed to identify clinically meaningful latent symptom profiles of adolescent MDD using the LPA. Given the heterogeneity of adolescent depressive presentations, we expected profiles to differ not only in overall symptom severity but also in the relative prominence of subjective sleep disturbance. Second, we compared the identified profiles across sociodemographic and recorded trauma/stressor‐related variables with the hypothesis that greater trauma/stressor exposure would be associated with the most clinically severe profile. Third, we examined whether neuroendocrine markers were associated with profile membership beyond trauma/stressor exposure. Given the exploratory nature of neuroendocrine profiling and the potential influence of developmental and physiological variability, specific hormonal associations were treated as hypothesis‐generating rather than confirmatory.

## 2. Methods

### 2.1. Participants and Procedure

This cross‐sectional study enrolled 711 adolescents admitted to the inpatient department of Hainan Anning Hospital between March 2024 and April 2025. The study protocol was approved by the Institutional Review Board of Hainan Medical University before recruitment, with renewal approval obtained in 2025 for the expanded study period and patient scope (Renewed Approval Number HYMLL‐2025‐159). All procedures were conducted in accordance with the Declaration of Helsinki. Written informed consent was obtained from parents or legal guardians, and written assent was obtained from adolescents before study enrollment.

Adolescents were eligible for inclusion if they (1) were aged 13–19 years; (2) were drug‐naïve, with no prior history of treatment with antidepressants or other psychotropic medications; and (3) met the diagnostic criteria for MDD.

To ensure diagnostic accuracy and reduce the risk of misclassification, particularly with bipolar disorder (BD) or psychotic disorders, MDD diagnoses were established according to DSM‐5 criteria by two independent attending‐level psychiatrists via comprehensive clinical interviews (DSM‐5 was utilized as it was the standardized diagnostic protocol integrated into the hospital’s system; core MDD criteria remain consistent with DSM‐5‐TR). Given the high risk of latent bipolarity in this demographic, specific screening for past manic or hypomanic episodes was conducted using detailed psychiatric and family histories obtained from both patients and their legal guardians. Furthermore, because participants were hospitalized, they underwent continuous clinical observation by psychiatric ward staff; any patient exhibiting spontaneous or treatment‐emergent hypomanic symptoms during hospitalization was reassessed diagnostically and excluded from the study.

Exclusion criteria included (1) the presence of any severe or unstable physical illness; (2) lifetime history of substance or alcohol abuse or dependence; (3) current or past diagnosis of a neurological disorder, significant head injury, or any comorbid psychiatric disorder with psychotic features; or (4) inability or refusal to provide written informed consent or assent.

After obtaining written informed consent and assent, specially trained psychiatric nurses administered the clinical questionnaires. Sociodemographic and clinical data were collected, cross‐validated with the hospital’s electronic medical record system to ensure accuracy, and subsequently de‐identified to protect participant confidentiality.

### 2.2. Clinical Assessments

To ensure temporal proximity to fasting blood sampling and minimize hospitalization‐related confounding, all clinical assessments (Hamilton depression rating scale [HDRS], Hamilton anxiety rating scale [HAMA], and Pittsburgh sleep quality index [PSQI]) were administered within 48 h of the admission.

#### 2.2.1. Depressive Symptoms

The 17‐item HDRS (HDRS‐17) was used to assess depressive symptom severity over the past week [27]. For the multidimensional analysis, we used the widely recognized seven‐factor structure: anxiety/somatization, weight, cognitive disturbance, diurnal variation, retardation, sleep disturbance, and guilt. In the present sample, the internal consistency for the HDRS‐17 total score was acceptable (Cronbach’s α = 0.716).

#### 2.2.2. Anxiety Symptoms

The 14‐item HAMA (HAMA‐14) was used to assess anxiety symptoms over the past week [28]. The scale included psychic and somatic anxiety dimensions. Internal consistency in the present sample was good (Cronbach’s α = 0.819).

#### 2.2.3. Sleep Quality

The PSQI was used to assess subjective sleep quality over the past month [29]. The PSQI comprises 19 self‐rated items grouped into seven component scores: subjective sleep quality, sleep latency, sleep duration, habitual sleep efficiency, sleep disturbances, use of sleeping medication, and daytime dysfunction. Each component was scored from 0 to 3, yielding a global score from 0 to 21. The internal consistency of the global score was acceptable in the present sample (Cronbach’s α = 0.715).

### 2.3. Neuroendocrine Measures

Serum neuroendocrine markers were measured on the first day of hospital admission for all participants. Blood samples were collected in the morning under fasting conditions according to established guidelines [30] and were processed according to the standard operating procedures (SOPs) of the hospital’s clinical laboratory. The following 11 hormones were assayed: follicle‐stimulating hormone (FSH), luteinizing hormone (LH), E2, T, prolactin (PRL), progesterone (P), thyroid‐stimulating hormone (TSH), total triiodothyronine (TT3), total thyroxine (TT4), free triiodothyronine (FT3), and free thyroxine (FT4).

### 2.4. Trauma/Stressor Exposure Assessment

In accordance with the hospital’s standard clinical evaluation protocol, information on trauma/stressor exposure was obtained through comprehensive psychiatric history‐taking rather than standardized psychometric questionnaires. Semi‐structured clinical interviews were administered by attending psychiatrists and routinely incorporated collateral information from legal guardians using a multi‐informant approach.

To quantify these clinical data, two independent trained researchers, blinded to patients’ symptom profiles, retrospectively reviewed and coded the electronic medical records for the presence of specific trauma/stressor categories. Inter‐rater reliability was substantial (Cohen’s κ = 0.818), and discrepancies were resolved through consensus discussion with a senior psychiatrist. This clinician‐administered approach was practical for acute inpatient settings but should be interpreted as clinical screening rather than a validated psychometric evaluation.

Trauma/stressor exposure was categorized into four primary types and coded as dichotomous variables (0 = absent and 1 = present): family trauma/stressor exposure, violent incidents, negative life events, and study‐related stress. A separate no obvious precipitating factor category was recorded descriptively but was not included in the cumulative trauma/stressor exposure index.

Family trauma/stressor exposure referred to adverse familial environments, including parental conflict, emotional neglect, or harsh parenting. Violent incidents included physical or verbal violence from others, peer victimization, or school bullying. Negative life events refer to significant adverse life stressors. Study‐related stress referred to substantial academic pressure or significant conflicts with teachers.

A cumulative trauma/stressor exposure index was constructed by summing the number of endorsed trauma/stressor categories. This index ranged from 0 to 4 and should be interpreted as a simple count of recorded exposure categories rather than as a validated dimensional measure of trauma burden, severity, chronicity, or developmental timing.

### 2.5. Sociodemographic and Clinical Data

Sociodemographic and clinical information was collected using a self‐report questionnaire, which was completed with the assistance of two child and adolescent psychiatric nurses. The questionnaire gathered data on age, sex, ethnicity, educational level, parental marital status, smoking and alcohol use history, perceived family support, suicidal ideation, history of suicide attempts, and length of hospital stay.

### 2.6. Statistical Analysis

All statistical analyses were performed using Python (Version 3.9) with its scientific computing libraries (pandas [31], NumPy [32], and scikit‐learn [33]) and IBM SPSS Statistics 23.0. All statistical tests were two‐tailed, and the significance level was set at *p* < 0.05.

#### 2.6.1. Descriptive Statistics

Descriptive statistics were calculated for all sociodemographic, clinical, and neuroendocrine variables. Continuous variables were presented as mean ± standard deviation (SD), while categorical variables were presented as frequencies and percentages (*n* and %).

#### 2.6.2. LPA and Sensitivity Analysis

To identify data‐driven symptom profiles, we conducted LPA [9, 10]. This approach identifies distinct symptom constellations rather than merely ranking participants along an overall severity continuum. The primary analysis used 16 continuous indicators, including seven HDRS‐17 factor scores, two HAMA‐14 factor scores, and seven PSQI component scores. Despite potential conceptual overlap among some symptom indicators, these indicators were included simultaneously in the primary analysis to capture the holistic and highly comorbid presentation of adolescent depression.

All 16 indicators were standardized as *z*‐scores before estimating 2‐ to 6‐profile solutions. Candidate models were evaluated using multiple criteria, including log‐likelihood, Akaike information criterion (AIC) [34], Bayesian information criterion (BIC) [35], sample‐size adjusted BIC (SABIC), entropy, average posterior probabilities, profile sizes, parsimony, and clinical interpretability [36]. Because information criteria may decrease with increasing model complexity, the final profile solution was not selected solely on the basis of the lowest AIC or BIC. Instead, preference was given to a solution that provided an appropriate balance of statistical fit, acceptable classification quality, adequate profile sizes, parsimony, and clinically meaningful symptom configurations.

To address potential measurement redundancy, HDRS and HAMA factor scores were recalculated after removing potentially overlapping sleep‐ and anxiety‐related items, including HDRS items 4–6, 10, and 11, and HAMA item 1. The remaining indicators were standardized, and the LPA was re‐estimated to examine whether the core profile pattern, particularly the PSQI‐elevated subjective sleep disturbance profile, remained identifiable after reducing overlap with PSQI sleep components. This analysis was intended to assess the consistency of the retained profile structure rather than to provide a definitive validation of profile stability.

As an exploratory robustness check, we further conducted a split‐sample stability analysis of the retained 3‐profile solution. The full sample was randomly divided into two halves, and the 3‐profile model was re‐estimated separately in each half. Profiles from each split‐sample solution were matched to the full‐sample profiles using maximum profile‐shape correlations based on standardized profile means. Higher profile‐shape correlations were interpreted as indicating greater similarity between the split‐sample and full‐sample profile structures.

#### 2.6.3. Inter‐Profile Group Comparisons

Differences across the identified profiles were examined. Continuous variables were analyzed using one‐way ANOVA [37] with Bonferroni post‐hoc tests [38] or the Kruskal–Wallis H test [39] when normality assumptions were violated. Categorical variables were analyzed using the chi‐square test [40].

#### 2.6.4. Hierarchical Multinomial Logistic Regression of Profile Correlates

To examine the associations of trauma/stressor and neuroendocrine variables with profile membership, hierarchical multinomial logistic regression was performed [41]. The three latent profiles served as the dependent variable, with Profile 1 as the reference group. Variables were entered in two blocks to evaluate the incremental information provided by neuroendocrine markers beyond trauma/stressor‐related variables [42]. Model 1 included the four dichotomous trauma/stressor variables: family trauma/stressor exposure, violent incidents, negative life events, and study‐related stress. As an exploratory analysis, Model 2 added the 11 neuroendocrine markers to examine potential neuroendocrine associations, which were interpreted with caution due to potential physiological and developmental confounding. Goodness‐of‐fit between nested models was compared using the LRT [43].

## 3. Results

### 3.1. Participant Characteristics

The final sample included 711 participants. Baseline demographic, clinical, and neuroendocrine characteristics are summarized in Table 1. The mean age was 14.87 ± 1.61 years, and the sample was predominantly female (*n* = 562, 79.0%) and of Han ethnicity (*n* = 663, 93.2%). The median length of hospital stay was 18.0 days (IQR: 12.0–27.0). Mean HDRS‐17 and HAMA‐14 scores were 29.53 ± 8.72 and 23.78 ± 8.20, respectively, and the median PSQI total score was 15.0 (IQR: 11.0–18.0). Suicidal ideation was reported by 560 participants (78.8%), and 149 participants (21.0%) had a history of suicide attempts.

**Table 1:** Baseline demographic, clinical, and neuroendocrine characteristics of participants (*N* = 711).

Characteristic	Value
Continuous variables (mean ± SD)	
Age (years)	14.87 ± 1.61
HDRS‐17 total score	29.53 ± 8.72
HAMA‐14 total score	23.78 ± 8.20
Skewed clinical and neuroendocrine indicators (median [IQR])	
Length of stay (days)	18.00 (12.00–27.00)
PSQI total score	15.00 (11.00–18.00)
PRL (ng/mL)	21.00 (15.00–26.00)
T (ng/dL)	20.35 (7.49–34.73)
E2 (pg/mL)	0.35 (0.21–1.11)
FSH (mIU/mL)	54.75 (40.14–83.76)
LH (mIU/mL)	5.41 (3.58–7.23)
P (ng/mL)	7.54 (4.49–12.00)
TSH (μIU/mL)	0.37 (0.20–0.66)
TT3 (ng/mL)	1.30 (1.10–2.06)
TT4 (μg/dL)	1.74 (1.62–2.08)
FT3 (pmol/L)	4.30 (3.90–4.60)
FT4 (pmol/L)	16.60 (15.05–17.80)
Categorical variables (*n* [%])	
Sex	
Female	562 (79.0%)
Male	149 (21.0%)
Ethnicity	
Han	663 (93.2%)
Li	43 (6.0%)
Other	5 (0.7%)
Education level	
Middle school	559 (78.6%)
Primary school	66 (9.3%)
College or higher	42 (5.9%)
Technical secondary school	34 (4.8%)
Illiterate	10 (1.4%)
Parental marital status	
Intact	677 (95.2%)
Divorced/separated	34 (4.8%)
History of smoking	
No	699 (98.3%)
Yes	11 (1.5%)
Unknown	1 (0.1%)
History of drinking	
No	704 (99.0%)
Yes	6 (0.8%)
Unknown	1 (0.1%)
Family support	
Moderate	645 (90.7%)
Good	53 (7.5%)
Poor	13 (1.8%)
Suicidal ideation	
Yes	560 (78.8%)
No	151 (21.2%)
History of suicide attempts	
No	562 (79.0%)
Yes	149 (21.0%)
Recorded trauma/stressor categories	
Family trauma/stressor exposure	291 (40.9%)
Negative life events	322 (45.2%)
Violent incidents	205 (28.8%)
Study‐related stress	364 (51.1%)
No obvious precipitating factor	89 (12.5%)
Cumulative trauma/stressor exposure index	
0	89 (12.5%)
1	232 (32.6%)
2	247 (34.7%)
3	116 (16.3%)
4	27 (3.7%)

Clinically, the participants showed high levels of depressive and anxiety symptoms. Normally distributed scores are reported as means ± SD, while skewed scores are reported as medians and interquartile ranges (IQRs). Specifically, the mean total scores for HDRS‐17 and HAMA‐14 were 29.53 ± 8.72 and 23.78 ± 8.20, respectively, whereas the median total score for PSQI was 15.0 (IQR: 11.0–18.0). A high proportion of adolescents reported suicidal ideation (*n* = 560, 78.8%) and a history of suicide attempts (*n* = 149, 21.0%).

### 3.2. LPA of Symptom Structure

To identify distinct symptom profiles, we conducted a LPA using 16 indicators from the HDRS, HAMA, and PSQI. We fitted models with 2–6 profiles. Detailed model fit indices for the primary analysis are provided in Table S1, and standardized mean plots for all candidate solutions are shown in Figure S1.

**Figure 1:**
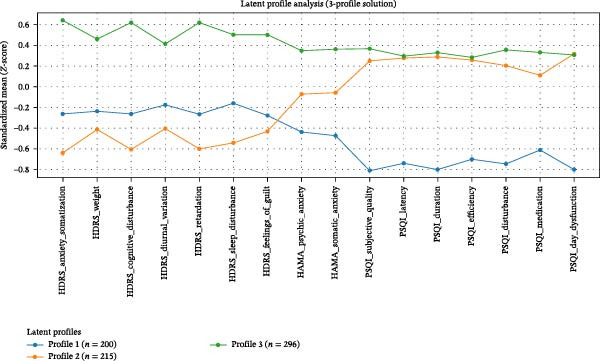
Standardized symptom means for the retained 3‐profile solution in the primary latent profile analysis.

The model fit indices did not unequivocally favor a single solution. Although some information criteria continued to improve with increasing model complexity, higher‐profile solutions tended to subdivide symptom variation into smaller and more indicator‐specific profiles, suggesting reduced parsimony. In contrast, the 2‐profile solution mainly represented a general low‐versus‐high symptom severity distinction and did not capture the clinically relevant pattern of relative PSQI‐assessed subjective sleep disturbance. Therefore, the 3‐profile solution was retained as the final model because it provided an appropriate balance of parsimony, clinical interpretability, adequate profile sizes, and acceptable classification quality. The standardized indicator means for the retained 3‐profile solution are provided in Table S4, and their overall pattern is illustrated in Figure 1.

#### 3.2.1. Profile 1: Low Overall Symptoms (*n* = 200, 28.1%)

This profile exhibited below‐average scores across all 16 symptom indicators, representing the group with the lowest overall symptom levels. Participants in this profile showed relatively low levels of depressive, anxious, and sleep‐related symptoms.

#### 3.2.2. Profile 2: PSQI‐Elevated Subjective Sleep Disturbance Profile (*n* = 215, 30.2%)

This profile was characterized by relatively low clinician‐rated depressive and anxiety symptom scores but relative elevations across self‐reported PSQI components, including subjective sleep quality, sleep latency, sleep duration, habitual sleep efficiency, sleep disturbances, use of sleep medication, and daytime dysfunction. Importantly, the HDRS sleep disturbance factor was not elevated in this profile relative to the high overall symptom profile. Therefore, this profile was labeled as the PSQI‐elevated subjective sleep disturbance profile, indicating that the sleep‐related pattern was driven primarily by PSQI‐assessed subjective sleep disturbance rather than by elevated clinician‐rated sleep disturbance.

#### 3.2.3. Profile 3: High Overall Symptoms (*n* = 296, 41.6%)

This profile showed above‐average scores across most HDRS, HAMA, and PSQI indicators, indicating the highest overall symptom levels. Compared with Profile 2, this profile showed higher clinician‐rated depressive and anxiety symptom levels, including higher HDRS sleep disturbance, while also showing elevated PSQI components.

A sensitivity analysis excluding potentially redundant HDRS sleep disturbance and anxiety/somatization indicators is summarized in Table S2 and Figure S2. The sensitivity analysis showed a broadly similar profile pattern to the primary analysis, including an identifiable PSQI‐elevated subjective sleep disturbance profile. However, information criteria did not unequivocally favor the 3‐profile solution as higher‐profile solutions showed lower information criteria but yielded smaller and more indicator‐specific profiles. Therefore, this analysis was interpreted as supporting a broadly similar profile pattern rather than providing definitive statistical confirmation of the 3‐profile model.

An exploratory split‐sample stability analysis of the retained 3‐profile solution is reported in Table S3. In the primary analysis, profile‐shape correlations between the full‐sample and split‐sample solutions were high across both random halves, ranging from 0.801 to 0.986. In the sensitivity analysis, Profiles 1 and 2 showed high shape similarity across both halves, whereas Profile 3 showed a more moderate similarity. These findings provide exploratory evidence of profile reproducibility in the primary analysis, with more limited stability in the sensitivity analysis.

### 3.3. Comparison of Characteristics Across Latent Profiles

The demographic, clinical, trauma/stressor‐related, and neuroendocrine characteristics of the three latent profiles are presented in Table 2. The three profiles did not differ significantly in age, length of hospital stay, sex, ethnicity, parental marital status, smoking history, or alcohol history.

**Table 2:** Comparison of demographic, clinical, trauma/stressor‐related, and neuroendocrine characteristics across the three latent profiles.

Variable	Profile 1 (*n* = 200)	Profile 2 (*n* = 215)	Profile 3 (*n* = 296)	Statistic	*p*
Demographics and behaviors					
Age (years)	15.02 ± 1.60	14.77 ± 1.70	14.86 ± 1.56	*F* = 1.25	0.288
Length of stay (days)	21.00 (15.00, 27.00)	20.00 (15.00, 26.00)	21.00 (15.90, 26.00)	*H* = 0.96	0.618
Male sex (*n* [%])	35 (17.5%)	42 (19.5%)	72 (24.3%)	*χ* ^2^ = 3.73	0.155
Han ethnicity (*n* [%])	191 (95.5%)	200 (93.0%)	272 (91.9%)	*χ* ^2^ = 2.77	0.597
Non‐intact family (*n* [%])	11 (5.5%)	11 (5.1%)	10 (3.4%)	*χ* ^2^ = 1.52	0.467
Smoking history (*n* [%])	0 (0.0%)	7 (3.3%)	4 (1.4%)	*χ* ^2^ = 8.74	0.068
Alcohol history (*n* [%])	1 (0.5%)	4 (1.9%)	1 (0.3%)	*χ* ^2^ = 5.24	0.264
Clinical Characteristics					
HDRS‐17 total score	26.00 (21.00, 31.00)	22.00 (18.00, 25.50)	36.00 (33.00, 41.00)	*H* = 442.96	**<0.** **001**
HAMA‐14 total score	19.00 (15.00, 23.00)	22.00 (17.00, 28.00)	26.00 (20.00, 32.25)	*H* = 84.14	**<0.001**
PSQI total score	9.00 (7.00, 11.00)	16.00 (14.00, 19.00)	17.00 (15.00, 20.00)	*H* = 403.28	**<0.001**
HDRS sleep disturbance factor	3.00 (3.00, 4.00)	3.00 (2.00, 4.00)	5.00 (3.00, 6.00)	*H* = 141.22	**<0.001**
Trauma/stressor exposure					
Cumulative trauma/stressor exposure index	1.00 (1.00, 2.00)	2.00 (1.00, 2.00)	2.00 (1.00, 3.00)	*H* = 22.53	**<** **0.001**
Family trauma/stressor exposure (*n* [%])	72 (36.0%)	65 (30.2%)	154 (52.0%)	*χ* ^2^ = 27.26	**<0.001**
Violent incidents (*n* [%])	38 (19.0%)	55 (25.6%)	112 (37.8%)	*χ* ^2^ = 22.23	**<0.001**
Life events (*n* [%])	87 (43.5%)	105 (48.8%)	130 (43.9%)	*χ* ^2^ = 1.58	0.455
Study‐related stress (*n* [%])	98 (49.0%)	110 (51.2%)	156 (52.7%)	*χ* ^2^ = 0.66	0.721
No obvious precipitating factor (*n* [%])	30 (15.0%)	29 (13.5%)	30 (10.1%)	*χ* ^2^ = 2.84	0.241
Neuroendocrine indicators					
PRL	18.74 (6.67, 32.24)	20.48 (10.34, 35.98)	21.24 (7.25, 35.30)	*H* = 2.35	0.309
E2	53.05 (35.14, 75.00)	54.48 (39.24, 90.05)	57.49 (45.03, 85.47)	*H* = 5.53	0.063
FSH	5.83 (4.00, 7.86)	5.12 (3.45, 6.81)	5.25 (3.42, 6.68)	*H* = 6.81	**0.033**
LH	8.03 (4.78, 12.47)	7.08 (4.26, 10.98)	7.60 (4.26, 12.16)	*H* = 1.48	0.476
P	0.38 (0.23, 0.65)	0.37 (0.18, 0.67)	0.37 (0.20, 0.65)	*H* = 1.16	0.559
T	0.31 (0.20, 0.69)	0.34 (0.20, 1.01)	0.39 (0.24, 2.28)	*H* = 10.13	**0.006**
TSH	1.30 (1.16, 1.91)	1.40 (1.08, 2.20)	1.30 (1.07, 2.00)	*H* = 0.79	0.673
TT3	1.73 (1.62, 2.01)	1.74 (1.62, 2.10)	1.74 (1.62, 2.10)	*H* = 0.55	0.761
TT4	96.15 (82.60, 103.00)	95.40 (83.75, 103.00)	97.50 (82.75, 103.00)	*H* = 0.33	0.846
FT4	16.60 (15.00, 17.60)	16.60 (14.87, 17.90)	16.60 (15.20, 17.70)	*H* = 0.40	0.817
FT3	4.83 (4.48, 5.19)	4.83 (4.38, 5.46)	4.83 (4.67, 5.46)	*H* = 6.69	**0.035**

*Note*: Values are presented as mean ± SD, median (interquartile range), or *n* (%). Statistical tests used were one‐way ANOVA, Kruskal–Wallis H test, Pearson’s chi‐square test, or Fisher’s exact test. Significant *p*‐values (<0.05) are highlighted in bold.

Abbreviatios: HAMA, Hamilton anxiety rating scale; HDRS, Hamilton depression rating scale; PSQI, Pittsburgh sleep quality index.

Significant overall group differences were observed for all clinical symptom measures, including HDRS‐17 total score, HAMA‐14 total score, PSQI total score, and the HDRS sleep disturbance factor, all *p* < 0.001. Profile 3 showed the highest clinician‐rated depressive symptom severity and the highest HDRS sleep disturbance factor, whereas Profile 2 showed elevated PSQI‐assessed subjective sleep problems despite lower overall clinician‐rated depressive severity compared with Profile 3.

Trauma/stressor exposure also differed across profiles. The cumulative trauma/stressor exposure index differed significantly among the three profiles, *H* = 22.53, *p* < 0.001. Significant group differences were observed for family trauma/stressor exposure, *χ^2^* = 27.26, *p* < 0.001, and violent incidents, *χ^2^* = 22.23, *p* < 0.001. In contrast, negative life events, study‐related stress, and no obvious precipitating factor did not differ significantly across profiles.

For neuroendocrine indicators, nominal overall differences were observed for FSH, *H* = 6.81, *p* = 0.033, T, *H* = 10.13, *p* = 0.006, and FT3, *H* = 6.69, *p* = 0.035. E2 showed a marginal difference across profiles, *H* = 5.53, *p* = 0.063. Given the number of neuroendocrine comparisons and the cross‐sectional design, these nominal differences were interpreted as exploratory.

### 3.4. Trauma/Stressor and Neuroendocrine Correlates of Symptom Profiles

Full hierarchical multinomial logistic regression results are provided in Table S5. Hierarchical multinomial logistic regression was conducted to examine associations of trauma/stressor and neuroendocrine variables with latent profile membership, with Profile 1 serving as the reference group. In the trauma/stressor‐only model, no trauma/stressor variable significantly distinguished Profile 2 from Profile 1. In contrast, family trauma/stressor exposure/stressor exposure and violent incidents were significantly associated with Profile 3 membership relative to Profile 1. Specifically, family trauma/stressor exposure/stressor exposure was associated with higher odds of Profile 3 membership, OR = 1.83, 95% CI [1.26, 2.67], *p* = 0.001, and violent incidents were also associated with higher odds of Profile 3 membership, OR = 2.49, 95% CI [1.62, 3.82], *p* < 0.001.

After adding neuroendocrine markers, the associations of family trauma/stressor exposure and violent incidents with Profile 3 membership remained significant. Family exposure/stressor exposure was associated with increased odds of Profile 3 membership, OR = 1.91, 95% CI [1.31, 2.80], *p* < 0.001, and violent incidents were associated with increased odds of Profile 3 membership, OR = 2.48, 95% CI [1.61, 3.82], *p* < 0.001. Among neuroendocrine markers, only higher standardized T showed a nominal association with greater odds of Profile 3 membership relative to Profile 1, OR = 1.24, 95% CI [1.00, 1.52], *p* = 0.045. FT3 showed a marginal association, OR = 1.37, 95% CI [0.94, 1.98], *p* = 0.098. No neuroendocrine marker was significantly associated with Profile 2 membership relative to Profile 1.

Model comparison indicated that adding neuroendocrine markers did not significantly improve model fit over the trauma/stressor‐only model, LR = 32.94, *df* = 22, *p* = 0.063 (Table S6). Moreover, AIC and BIC were higher in the expanded model, suggesting that the more parsimonious trauma/stressor‐only model provided a better overall balance between fit and complexity.

## 4. Discussion

Using LPA in 711 drug‐naïve adolescents with MDD, we retained a 3‐profile solution that provided a parsimonious and clinically interpretable representation of symptom heterogeneity. The profiles were characterized as low overall symptoms (Profile 1; *n* = 200, 28.1%), PSQI‐elevated subjective sleep disturbance (Profile 2; *n* = 215, 30.2%), and high overall symptoms (Profile 3; *n* = 296, 41.6%). This structure suggests that adolescent depression is not only differentiated by overall symptom severity but also by the relative prominence of PSQI‐assessed subjective sleep disturbance. In this sense, the present findings highlight the potential clinical relevance of multidimensional symptom profiling in adolescent MDD, particularly in drug‐naïve patients, for whom symptom patterns are less likely to be confounded by medication effects.

The PSQI‐elevated subjective sleep disturbance profile was characterized by relatively low clinician‐rated depressive and anxiety symptom severity but modest elevations across self‐reported PSQI sleep components. In contrast, the clinician‐rated HDRS sleep disturbance factor was higher in the high overall symptom profile than in Profile 2. Therefore, Profile 2 should be interpreted primarily as a profile characterized by relative elevations in PSQI‐assessed past‐month subjective sleep disturbance rather than as evidence of a sleep‐specific subtype consistently captured across clinician‐rated and self‐reported instruments or as the group with the most severe clinician‐rated sleep disturbance. This discrepancy may reflect differences in instrument content and assessment windows: HDRS sleep‐related items assess clinician‐rated sleep disturbance symptoms within the context of depressive symptom severity during the past week, whereas the PSQI captures multidimensional subjective sleep problems over the past month, including sleep quality, latency, duration, efficiency, disturbances, medication use, and daytime dysfunction [29]. These findings underscore the potential importance of incorporating comprehensive sleep assessment in pediatric psychiatric settings.

The PSQI components in Profile 2 approached the levels observed in the high overall symptom profile despite lower clinician‐rated affective and anxiety symptoms. This pattern is consistent with prior evidence suggesting that self‐reported sleep problems may be partially dissociable from overall depressive symptom severity in mood disorders [44, 45]. In adolescents undergoing normative circadian changes, sleep disturbances and affective symptoms may represent related but distinguishable dimensions relevant to emotion regulation and stress responses [46–48]. Therefore, the PSQI‐elevated subjective sleep disturbance profile may help identify adolescents whose self‐reported sleep complaints warrant careful clinical assessment, even when overall clinician‐rated depressive severity is not the highest. Sleep‐focused assessment and supportive clinical management may complement standard depression care for adolescents reporting prominent subjective sleep complaints [49]. However, because this profile was primarily PSQI‐driven and was not supported by a parallel elevation in HDRS sleep disturbance, its interpretation as a distinct sleep‐related clinical phenotype should remain preliminary.

Trauma/stressor exposure provided an important external clinical context for the retained profile solution. The high overall symptom profile showed the highest cumulative count of recorded trauma/stressor categories and the highest proportions of family trauma/stressor exposure and violent incidents. In unadjusted comparisons, family trauma/stressor exposure was reported by 52.0% of adolescents in Profile 3, compared with 36.0% in Profile 1 and 30.2% in Profile 2. Violent incidents were reported by 37.8% of Profile 3, compared with 19.0% and 25.6% in Profiles 1 and 2, respectively. In the adjusted multinomial regression model, family trauma/stressor exposure and violent incidents remained significantly associated with Profile 3 membership relative to Profile 1. These findings suggest that interpersonal and family‐related adversity may be particularly relevant to the high overall symptom profile.

From a theoretical perspective, interpersonal trauma and adversity have been linked to dysregulation within the HPA and HPG axes [50–52], as well as broader alterations in stress‐response systems, emotion regulation, threat processing, and inflammatory pathways [53–55]. The present findings are consistent with this literature in that the profile characterized by the high overall symptom profile also showed the highest trauma/stressor exposure. However, given the strictly cross‐sectional design, we cannot determine whether trauma/stressor exposure temporally preceded the emergence of this symptom profile, whether adolescents with more severe symptoms were more likely to report or receive documentation of trauma/stressor exposure, or whether both reflect broader developmental vulnerability. Therefore, trauma/stressor exposure should be interpreted as a clinically relevant correlate of the high overall symptom profile rather than as a causal determinant of profile membership.

The contribution of neuroendocrine markers was less clear. In unadjusted comparisons, FSH, *T*, and FT3 showed nominally significant differences across profiles, and E2 showed a marginal difference. However, these between‐profile differences did not translate into strong independent associations in the adjusted regression model. After simultaneous adjustment for trauma/stressor variables and other neuroendocrine markers, only higher standardized T showed a modest association with Profile 3 membership relative to Profile 1, *OR* = 1.24, 95% *CI* [1.00, 1.52], *p* = 0.045, whereas standardized FT3 showed only a marginal association, *OR* = 1.37, 95% *CI* [0.94, 1.98], *p* = 0.098. No neuroendocrine marker was significantly associated with Profile 2 membership relative to Profile 1. Thus, although individual neuroendocrine indicators showed preliminary associations with symptom profiles, their incremental explanatory value was limited.

The model comparison further supported the limited incremental value of neuroendocrine markers. Adding the neuroendocrine marker block did not significantly improve model fit over the trauma‐only model, *LR* = 32.94, *df* = 22, *p* = 0.063. Moreover, AIC increased from 1510.93 to 1521.98, and BIC increased from 1556.59 to 1668.12 after adding neuroendocrine indicators, suggesting that the expanded model did not provide a better balance between explanatory gain and model complexity. Because adding the neuroendocrine marker block did not significantly improve the global fit, the modest association between T and Profile 3 should be interpreted cautiously. Rather than indicating a specific or clinically actionable biomarker, this finding may reflect broader pubertal, stress‐related, metabolic, or developmental variations.

These individual neuroendocrine associations should therefore be regarded as exploratory and hypothesis‐generating. Although prior studies have linked gonadal hormones, including E2, to stress‐related neural processes [56] and thyroid hormone alterations to adrenergic and cognitive symptoms of depression [57–60], the current results do not provide evidence for a robust neuroendocrine signature that clearly differentiates latent symptom profiles. This may be partly attributable to the modest effect sizes of individual neuroendocrine markers, the number of neuroendocrine markers examined, potential intercorrelations among neuroendocrine systems, and the lack of repeated measurements. Furthermore, because hormone levels are influenced by the pubertal stage, menstrual‐cycle phase, body composition, circadian timing, and acute stress state, single‐time‐point neuroendocrine assessments may not adequately capture stable or trait‐like neuroendocrine functioning in adolescents [61]. Future studies with repeated neuroendocrine sampling, pubertal staging, menstrual‐cycle information, and larger longitudinal cohorts are needed to determine whether neuroendocrine variation contributes to the development or maintenance of specific depressive symptom profiles.

Several limitations should be acknowledged. First, the cross‐sectional, single‐center design limits causal inference and may restrict the generalizability of the findings to community samples, outpatient populations, or adolescents with different illness severity. Longitudinal research is needed to elucidate the temporal dynamics among trauma/stressor exposure, neuroendocrine changes, sleep disturbance, and symptom emergence. Second, trauma/stressor assessment in this study represents a major limitation. Trauma/stressor exposure was retrospectively retrieved from clinical records and coded dichotomously as present or absent rather than assessed using validated dimensional psychometric instruments such as the Childhood Trauma Questionnaire [62]. Because of this, we were unable to capture key dimensions of trauma/stressor exposure, including chronicity, objective severity, developmental timing, subjective distress, and cumulative exposure load. Furthermore, our cumulative trauma/stressor exposure index treated heterogeneous categories, such as chronic family‐related adversity and episodic academic stress, as equivalent. This simplified summation may obscure the differential severity, clinical salience, and developmental impact of different trauma/stressor types. Therefore, our findings should not be interpreted as representing a validated dimensional “trauma burden,” and future research should use standardized psychometric scales to disentangle specific trauma/stressor characteristics.

Third, although the sample was drug‐naïve, reducing confounding by psychotropic medication, we were unable to control for several important physiological covariates. These included the pubertal stage, menstrual‐cycle phase in female participants, body mass index, circadian timing of blood collection, acute stress state, and sex‐specific hormonal reference ranges. These unmeasured factors may have contributed to variability in neuroendocrine measures and may partly explain the limited incremental value of neuroendocrine markers in the regression models. Fourth, because multiple neuroendocrine indicators were examined, some nominally significant neuroendocrine associations may reflect type I errors. This concern is particularly relevant because the global model improvement after adding neuroendocrine markers was only marginal and did not reach conventional statistical significance. Thus, the neuroendocrine findings should be interpreted as preliminary rather than confirmatory.

Finally, although the retained 3‐profile solution was clinically interpretable and supported by adequate profile sizes, LPA remains a data‐driven method. The profiles should therefore be viewed as empirically derived symptom patterns rather than fixed diagnostic subtypes. Replication in independent samples, ideally using longitudinal designs and external clinical outcomes, is necessary to determine whether these profiles are stable over time and whether they predict the treatment response, recurrence risk, or functional impairment.

## 5. Conclusion

In conclusion, this study contributes to a more nuanced understanding of adolescent MDD by identifying three data‐driven and clinically interpretable profiles: low overall symptoms, a PSQI‐elevated subjective sleep disturbance profile, and high overall symptoms. The PSQI‐elevated subjective sleep disturbance profile should be interpreted as reflecting primarily PSQI‐assessed past‐month subjective sleep problems rather than evidence of a sleep‐specific subtype consistently captured across instruments. These profiles were characterized by distinct multidimensional symptom presentations and clinically relevant differences in trauma/stressor exposure, with family trauma/stressor exposure and violent incidents showing the most consistent associations with the high overall symptom profile. In contrast, neuroendocrine markers showed only limited and exploratory associations with profile membership, and adding them did not significantly improve model fit beyond trauma‐/stressor‐related variables. Rather than representing definitive biological signatures, these neuroendocrine findings should be interpreted as preliminary hypothesis‐generating signals. Future studies using longitudinal designs, larger independent cohorts, repeated neuroendocrine assessments, standardized trauma measures, and dedicated sleep assessments are needed to determine whether these biopsychosocial patterns can be replicated, clinically validated, and translated into targeted assessment or intervention strategies.

## Author Contributions

Yan He and Yin Lin were responsible for the overall design and promotion of the research. Mengmeng Yang and Xinyi Cao managed the data and the project. Jianyu Liu conducted the data analysis and wrote the manuscript for the research.

## Funding

The authors received no specific funding for this work.

## Disclosure

All the authors approved the final manuscript as submitted and agreed to be accountable for all aspects of the work. After using this tool, the authors reviewed and edited the content as needed and take full responsibility for the content of the publication.

## Consent

All participants provided a written informed consent for publication.

## Conflicts of Interest

The authors declare no conflicts of interest.

## Supporting Information

Additional supporting information can be found online in the Supporting Information section.

## Supporting information


**Supporting Information** The Supporting Information provides additional information on model selection, profile interpretation, and robustness checks for the latent profile analyses. Table S1 presents the model fit indices for the 2‐ to 6‐profile solutions in the primary latent profile analysis. Figure S1 displays the standardized mean profiles for these candidate solutions. Table S2 presents the model fit indices for the 2‐ to 6‐profile solutions in the sensitivity analysis excluding potentially overlapping indicators, and Supporting Figure S2 displays the corresponding standardized mean profiles. Table S3 reports the exploratory split‐sample stability analysis of the retained 3‐profile solution. Table S4 provides the standardized indicator means for the retained 3‐profile primary solution. Table S5 presents the hierarchical multinomial logistic regression results predicting latent profile membership. Table S6 presents model comparison for hierarchical multinomial logistic regression.

## Data Availability

The data that support the findings of this study are available from Anning Hospital, Hainan Province. Restrictions apply to the availability of these data, which were used under license for this study. Data are available from the authors with the permission of Anning Hospital, Hainan Province.
