# Supplementary material for: Latent Symptom Profiles in Adolescents With Major Depressive Disorder: Subjective Sleep Disturbance and Biopsychosocial Correlates
Source: Depress Anxiety. 2026 Jul 24;2026:9254081. doi: 10.1155/da/9254081 (PMC13397475; doi:10.1155/da/9254081)
Supplement: Supplementary file 1 — Supporting Information The Supporting Information provides additional information on model selection, profile interpretation, and robustness checks for the latent profile analyses. Table S1 presents the model fit indices for the 2‐ to 6‐profile solutions in the primary latent profile analysis. Figure S1 displays the standardized mean profiles for these candidate solutions. Table S2 presents the model fit indices for the 2‐ to 6‐profile solutions in the sensitivity analysis excluding potentially overlapping indicators, and Supporting Figure S2 displays the corresponding standardized mean profiles. Table S3 reports the exploratory split‐sample stability analysis of the retained 3‐profile solution. Table S4 provides the standardized indicator means for the retained 3‐profile primary solution. Table S5 presents the hierarchical multinomial logistic regression results predicting latent profile membership. Table S6 presents model comparison for hierarchical multinomial logistic regression. [file DA-2026-9254081-s001.docx]

Supplementary latent profile analyses

To provide a more comprehensive and transparent evaluation of model selection and profile stability, we estimated 2- to 6-profile solutions in both the primary latent profile analysis and a sensitivity analysis excluding potentially redundant HDRS sleep disturbance and anxiety/somatization indicators. For each candidate solution, we report log-likelihood, AIC, BIC, sample-size adjusted BIC, entropy, average posterior probability range, smallest profile size, and profile sizes. Standardized mean plots were generated for all candidate solutions using a common y-axis scale to facilitate direct visual comparison. We also conducted an exploratory split-sample stability analysis of the retained 3-profile solution by randomly dividing the sample into two halves and comparing profile shapes in each half with the full-sample 3-profile solution. All candidate LPA models were estimated using multiple random starts to reduce the risk of local maxima, and the best log-likelihood solution was checked for replication.

.

**Supplementary Table S1. Model fit indices for the 2- to 6-profile solutions in the primary latent profile analysis**

| **Model** | **Log-likelihood** | **AIC** | **BIC** | **SABIC** | **Entropy** | **APP range** | **Smallest Profile,**  **n (%)** | **Profile sizes** |
| --- | --- | --- | --- | --- | --- | --- | --- | --- |
| 2-profile | -15353.90 | 30837.72 | 31134.56 | 30928.17 | 0.842 | 0.948–0.963 | 302 (42.5%) | 302, 409 |
| 3-profile | -14942.00 | 30080.01 | 30527.54 | 30216.37 | 0.856 | 0.913–0.953 | 200 (28.1%) | 200, 215, 296 |
| 4-profile | -14777.30 | 29816.56 | 30414.80 | 29998.84 | 0.842 | 0.895–0.928 | 118 (16.6%) | 118, 208, 171, 214 |
| 5-profile | -12810.70 | 25949.35 | 26698.28 | 26177.54 | 0.942 | 0.914–0.993 | 72 (10.1%) | 161, 87, 276, 115, 72 |
| 6-profile | -12304.70 | 25003.36 | 25902.99 | 25277.47 | 0.937 | 0.932–0.985 | 50 (7.0%) | 185, 93, 66, 218, 99, 50 |

**Note. AIC = Akaike information criterion; BIC = Bayesian information criterion; SABIC = sample-size adjusted Bayesian information criterion; APP = average posterior probability. APP range indicates the minimum and maximum average posterior probabilities across profiles within each candidate solution. Entropy reflects classification certainty, with higher values indicating clearer classification. Profiles were ordered according to the overall standardized symptom level within each model. Although information criteria decreased with increasing profile number, the retained solution was selected based on a balance of statistical fit, classification quality, profile size, parsimony, and clinical interpretability.**

Interpretation of competing solutions in the primary analysis. In the primary analysis, information criteria continued to decrease from the 2- to 6-profile solutions, with the lowest AIC, BIC, and SABIC observed for the 6-profile solution. However, the 2-profile solution mainly represented a general low-versus-high symptom severity distinction and did not separate a clinically meaningful sleep-disturbance-dominant subgroup. The 3-profile solution separated participants into a low overall symptom profile, a PSQI-elevated subjective sleep disturbance profile, and a high overall symptom profile. Providing a parsimonious structure directly aligned with the clinical aims of the study. The 4-profile solution further subdivided severity and sleep-related differences. The 5- and 6-profile solutions yielded lower information criteria but introduced smaller and more indicator-specific profiles, suggesting reduced clinical parsimony and greater risk of over-extraction. Therefore, the 3-profile solution was retained as the most parsimonious and clinically interpretable solution with adequate profile sizes and acceptable classification quality, rather than as the statistically optimal solution according to information criteria alone.

.

**
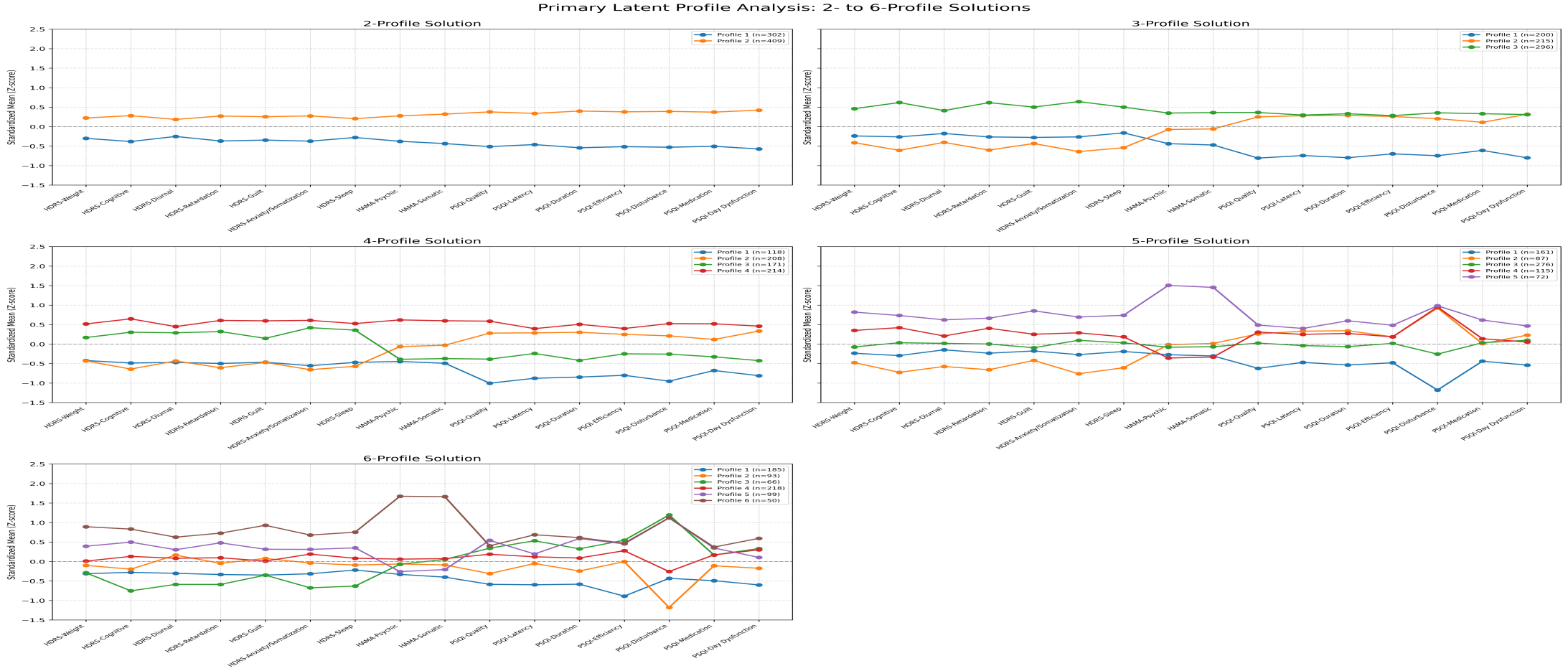
**

**Supplementary Figure S1. Standardized mean profiles for the 2- to 6-profile solutions in the primary latent profile analysis. The primary analysis included 16 standardized HDRS, HAMA, and PSQI indicators. The horizontal dashed line represents the sample mean of zero after standardization. All panels were plotted using a common y-axis scale from -1.5 to 2.5. Although higher-profile solutions showed lower information criteria, the 3-profile solution displayed a more parsimonious and clinically interpretable pattern consisting of a low overall symptom profile, a PSQI-elevated subjective sleep disturbance profile, and a high overall symptom profile.**

**Supplementary Table S2. Model fit indices for the 2- to 6-profile solutions in the sensitivity latent profile analysis**

| **Model** | **Log-likelihood** | **AIC** | **BIC** | **SABIC** | **Entropy** | **APP range** | **Smallest Profile,**  **n (%)** | **Profile sizes** |
| --- | --- | --- | --- | --- | --- | --- | --- | --- |
| 2-profile | -13392.40 | 26898.85 | 27159.15 | 26978.16 | 0.841 | 0.941–0.963 | 288 (40.5%) | 288, 423 |
| 3-profile | -13120.40 | 26412.90 | 26805.63 | 26532.56 | 0.838 | 0.897–0.950 | 179 (25.2%) | 208, 179, 324 |
| 4-profile | -12978.90 | 26187.79 | 26712.96 | 26347.81 | 0.817 | 0.867–0.925 | 104 (14.6%) | 104, 174, 178, 255 |
| 5-profile | -9809.11 | 19906.22 | 20563.82 | 20106.58 | 0.982 | 0.922–0.996 | 40 (5.6%) | 199, 283, 43, 146, 40 |
| 6-profile | -9549.72 | 19445.45 | 20235.48 | 19686.17 | 0.927 | 0.838–0.987 | 25 (3.5%) | 182, 263, 100, 109, 25, 32 |

**Note. The sensitivity analysis excluded potentially redundant HDRS sleep disturbance and anxiety/somatization indicators. AIC = Akaike information criterion; BIC = Bayesian information criterion; SABIC = sample-size adjusted Bayesian information criterion; APP = average posterior probability. APP range indicates the minimum and maximum average posterior probabilities across profiles within each candidate solution. Entropy reflects classification certainty, with higher values indicating clearer classification. Profile sizes are presented according to the profile numbering used in each k-profile solution. Profiles were ordered by the overall standardized symptom level within each model. The sensitivity analysis was used to examine whether the main profile pattern was robust to the exclusion of overlapping indicators.**

Interpretation of competing solutions in the sensitivity analysis. In the sensitivity analysis excluding potentially redundant HDRS sleep disturbance and anxiety/somatization indicators, the 2-profile solution again reflected a low-versus-high severity distinction. The 3-profile solution showed a broadly similar structure to the primary analysis, PSQI-elevated subjective sleep disturbance profile. The 4-profile solution further subdivided the low-symptom and sleep-related profiles, whereas the 5- and 6-profile solutions yielded smaller profiles with sharper indicator-specific elevations, suggesting reduced clinical parsimony. These findings were therefore interpreted as indicating a broadly similar but not unequivocally superior 3-profile pattern after excluding overlapping indicators.

**
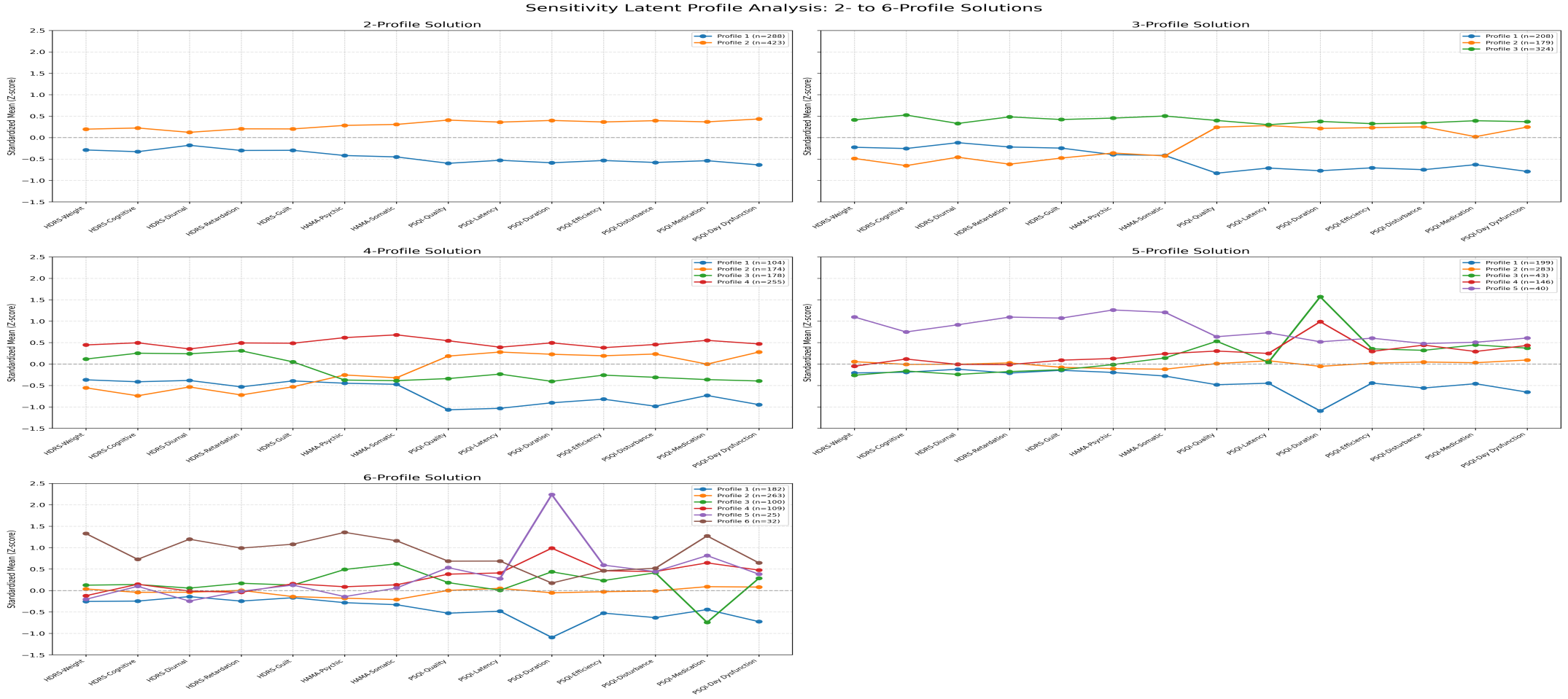
**

**Supplementary Figure S2. Standardized mean profiles for the 2- to 6-profile solutions in the sensitivity latent profile analysis. This analysis excluded potentially redundant HDRS sleep disturbance and anxiety/somatization indicators. The horizontal dashed line represents the sample mean of zero after standardization. All panels were plotted using a common y-axis scale from -1.5 to 2.5. The 3-profile solution showed a broadly similar structure to the primary analysis, including an identifiable PSQI-elevated subjective sleep disturbance profile. Higher-profile solutions produced smaller and more indicator-specific profiles, suggesting reduced clinical parsimony.**

**Supplementary Table S3. Exploratory split-sample stability analysis of the 3-profile solution**

| **Analysis** | **Split** | **Full-sample profile** | **Matched split-sample profile** | **Profile-shape correlation** |
| --- | --- | --- | --- | --- |
| Primary analysis | Half A | 1 | 1 | 0.980 |
| Primary analysis | Half A | 2 | 2 | 0.982 |
| Primary analysis | Half A | 3 | 3 | 0.801 |
| Primary analysis | Half B | 1 | 1 | 0.952 |
| Primary analysis | Half B | 2 | 2 | 0.986 |
| Primary analysis | Half B | 3 | 3 | 0.823 |
| Sensitivity analysis | Half A | 1 | 1 | 0.972 |
| Sensitivity analysis | Half A | 2 | 2 | 0.960 |
| Sensitivity analysis | Half A | 3 | 3 | 0.583 |
| Sensitivity analysis | Half B | 1 | 1 | 0.951 |
| Sensitivity analysis | Half B | 2 | 2 | 0.990 |
| Sensitivity analysis | Half B | 3 | 3 | 0.579 |

**Note. The split-sample stability analysis was conducted as an exploratory robustness check. The sample was randomly divided into two halves, and the 3-profile solution was re-estimated separately in each half. Split-sample profiles were matched to the full-sample profiles using maximum profile-shape correlations based on standardized profile means. In the primary analysis, profile-shape correlations were high across both split halves, supporting the stability of the 3-profile structure. In the sensitivity analysis, Profiles 1 and 2 showed high shape similarity, whereas Profile 3 showed weaker shape similarity, indicating partial stability after excluding overlapping indicators.The sample was randomly divided into two halves of approximately equal size.**

**Supplementary Table S4. Standardized indicator means for the retained 3-profile solution**

| **Indicator** | **Profile 1: Low overall symptoms** | **Profile 2: PSQI-elevated subjective sleep disturbance** | **Profile 3: High overall symptoms** |
| --- | --- | --- | --- |
| HDRS anxiety/somatization | -0.254 | -0.649 | 0.644 |
| HDRS weight loss | -0.253 | -0.407 | 0.470 |
| HDRS cognitive disturbance | -0.273 | -0.602 | 0.624 |
| HDRS diurnal variation | -0.148 | -0.433 | 0.414 |
| HDRS retardation | -0.260 | -0.605 | 0.617 |
| HDRS sleep disturbance | -0.166 | -0.553 | 0.513 |
| HDRS feelings of guilt | -0.262 | -0.435 | 0.497 |
| HAMA psychic anxiety | -0.437 | -0.068 | 0.360 |
| HAMA somatic anxiety | -0.458 | -0.057 | 0.367 |
| PSQI subjective sleep quality | -0.774 | 0.265 | 0.363 |
| PSQI sleep latency | -0.717 | 0.287 | 0.307 |
| PSQI sleep duration | -0.790 | 0.311 | 0.342 |
| PSQI habitual sleep efficiency | -0.687 | 0.283 | 0.288 |
| PSQI sleep disturbances | -0.728 | 0.222 | 0.361 |
| PSQI use of sleep medication | -0.616 | 0.140 | 0.339 |
| PSQI daytime dysfunction | -0.771 | 0.335 | 0.311 |

**Note. Values are standardized means of the indicators used in the retained 3-profile primary latent profile solution. Higher values indicate greater symptom severity or poorer sleep. Profile labels were revised to reflect the observed indicator pattern. Profile 2 showed relative elevations across PSQI components but did not show an elevated HDRS sleep disturbance factor relative to the high overall symptom profile; therefore, it was labeled as the PSQI-elevated subjective sleep disturbance profile rather than an insomnia-dominant profile.**

**Supplementary Table S5. Hierarchical Multinomial Logistic Regression Predicting Latent Profile Membership**

Reference group: Profile 1, Low Overall Symptoms.

| **Model** | **Comparison** | ***Predictor*** | ***OR*** | ***95% CI*** | ***p*** |
| --- | --- | --- | --- | --- | --- |
| Model 1: Trauma only | Profile 2 vs Profile 1 | Family trauma | 0.75 | 0.50–1.13 | .167 |
| Model 1: Trauma only | Profile 2 vs Profile 1 | Violent incidents | 1.49 | 0.93–2.38 | .096 |
| Model 1: Trauma only | Profile 2 vs Profile 1 | Negative Life Events | 1.25 | 0.85–1.84 | .264 |
| Model 1: Trauma only | Profile 2 vs Profile 1 | Study-related stress | 1.09 | 0.74–1.60 | .674 |
| Model 1: Trauma only | Profile 3 vs Profile 1 | Family trauma | 1.83 | 1.26–2.67 | .001 |
| Model 1: Trauma only | Profile 3 vs Profile 1 | Violent incidents | 2.49 | 1.62–3.82 | < .001 |
| Model 1: Trauma only | Profile 3 vs Profile 1 | Negative Life Events | 0.97 | 0.67–1.41 | .876 |
| Model 1: Trauma only | Profile 3 vs Profile 1 | Study-related stress | 1.14 | 0.79–1.64 | .492 |
| Model 2: Trauma + Endocrine | Profile 2 vs Profile 1 | Family trauma | 0.74 | 0.48–1.12 | .148 |
| Model 2: Trauma + Endocrine | Profile 2 vs Profile 1 | Violent incidents | 1.41 | 0.88–2.27 | .156 |
| Model 2: Trauma + Endocrine | Profile 2 vs Profile 1 | Negative Life Events | 1.23 | 0.83–1.82 | .304 |
| Model 2: Trauma + Endocrine | Profile 2 vs Profile 1 | Study-related stress | 1.10 | 0.74–1.63 | .633 |
| Model 2: Trauma + Endocrine | Profile 2 vs Profile 1 | PRL_z | 2.79 | 0.24–32.78 | .415 |
| Model 2: Trauma + Endocrine | Profile 2 vs Profile 1 | E2_z | 1.05 | 0.81–1.36 | .714 |
| Model 2: Trauma + Endocrine | Profile 2 vs Profile 1 | FSH_z | 1.01 | 0.80–1.27 | .942 |
| Model 2: Trauma + Endocrine | Profile 2 vs Profile 1 | LH_z | 0.90 | 0.61–1.32 | .594 |
| Model 2: Trauma + Endocrine | Profile 2 vs Profile 1 | P_z | 1.11 | 0.87–1.41 | .416 |
| Model 2: Trauma + Endocrine | Profile 2 vs Profile 1 | T_z | 1.14 | 0.91–1.43 | .240 |
| Model 2: Trauma + Endocrine | Profile 2 vs Profile 1 | TSH_z | 0.93 | 0.71–1.21 | .593 |
| Model 2: Trauma + Endocrine | Profile 2 vs Profile 1 | TT3_z | 1.79 | 0.02–157.42 | .799 |
| Model 2: Trauma + Endocrine | Profile 2 vs Profile 1 | TT4_z | 1.12 | 0.76–1.66 | .563 |
| Model 2: Trauma + Endocrine | Profile 2 vs Profile 1 | FT4_z | 0.84 | 0.61–1.15 | .282 |
| Model 2: Trauma + Endocrine | Profile 2 vs Profile 1 | FT3_z | 1.04 | 0.70–1.53 | .857 |
| Model 2: Trauma + Endocrine | Profile 3 vs Profile 1 | Family trauma | 1.91 | 1.31–2.80 | < .001 |
| Model 2: Trauma + Endocrine | Profile 3 vs Profile 1 | Violent incidents | 2.48 | 1.61–3.82 | < .001 |
| Model 2: Trauma + Endocrine | Profile 3 vs Profile 1 | Negative Life Events | 0.98 | 0.67–1.43 | .926 |
| Model 2: Trauma + Endocrine | Profile 3 vs Profile 1 | Study-related stress | 1.11 | 0.77–1.62 | .576 |
| Model 2: Trauma + Endocrine | Profile 3 vs Profile 1 | PRL_z | 2.28 | 0.20–26.67 | .511 |
| Model 2: Trauma + Endocrine | Profile 3 vs Profile 1 | E2_z | 1.10 | 0.87–1.41 | .422 |
| Model 2: Trauma + Endocrine | Profile 3 vs Profile 1 | FSH_z | 0.84 | 0.66–1.06 | .134 |
| Model 2: Trauma + Endocrine | Profile 3 vs Profile 1 | LH_z | 1.22 | 0.90–1.66 | .207 |
| Model 2: Trauma + Endocrine | Profile 3 vs Profile 1 | P_z | 0.99 | 0.78–1.26 | .952 |
| Model 2: Trauma + Endocrine | Profile 3 vs Profile 1 | T_z | 1.24 | 1.00–1.52 | .045 |
| Model 2: Trauma + Endocrine | Profile 3 vs Profile 1 | TSH_z | 1.06 | 0.89–1.26 | .526 |
| Model 2: Trauma + Endocrine | Profile 3 vs Profile 1 | TT3_z | 0.08 | 0.00–6.53 | .261 |
| Model 2: Trauma + Endocrine | Profile 3 vs Profile 1 | TT4_z | 1.19 | 0.81–1.74 | .382 |
| Model 2: Trauma + Endocrine | Profile 3 vs Profile 1 | FT4_z | 1.00 | 0.85–1.18 | .982 |
| Model 2: Trauma + Endocrine | Profile 3 vs Profile 1 | FT3_z | 1.37 | 0.94–1.98 | .098 |

**Notes. Multinomial logistic regression was used with Profile 1, the Low Overall Symptoms profile, as the reference group. Trauma variables were coded as yes versus no. Endocrine indicators were standardized, so odds ratios for endocrine variables represent the change in odds associated with a one-standard-deviation increase in the corresponding marker. OR = odds ratio; CI = confidence interval; PRL = prolactin; E2 = estradiol; FSH = follicle-stimulating hormone; LH = luteinizing hormone; P = progesterone; T = testosterone; TSH = thyroid-stimulating hormone; TT3 = total triiodothyronine; TT4 = total thyroxine; FT4 = free thyroxine; FT3 = free triiodothyronine.**

**Supplementary Table S6. Model Comparison for Hierarchical Multinomial Logistic Regression**

| **Model** | **Log-likelihood** | **AIC** | **BIC** | ***df*** | **LR statistic** | ***p*** |
| --- | --- | --- | --- | --- | --- | --- |
| Model 1: Trauma only | -745.46 | 1510.93 | 1556.59 | 8 | — | — |
| Model 2: Trauma + Endocrine | -728.99 | 1521.98 | 1668.12 | 30 | — | — |
| LRT: Model 2 vs Model 1 | — | — | — | 22 | 32.94 | .063 |

**Notes. Model 1 included trauma exposure variables only. Model 2 additionally included standardized endocrine indicators. The likelihood-ratio test indicated that adding endocrine markers did not significantly improve model fit over the trauma-only model. AIC and BIC were higher in Model 2, further supporting the more parsimonious trauma-only model.**
